# Cloning and Functional Characterization of Novel Variants and Tissue-Specific Expression of Alternative Amino and Carboxyl Termini of Products of *Slc4a10*

**DOI:** 10.1371/journal.pone.0055974

**Published:** 2013-02-07

**Authors:** Ying Liu, Deng-Ke Wang, De-Zhi Jiang, Xue Qin, Zhang-Dong Xie, Qing K. Wang, Mugen Liu, Li-Ming Chen

**Affiliations:** 1 Department of Biophysics and Molecular Physiology, Key Laboratory of Molecular Biophysics of Ministry of Education, Huazhong University of Science & Technology School of Life Science & Technology, Wuhan, Hubei, China; 2 Department of Physiology and Biophysics, Case Western Reserve University School of Medicine, Cleveland, Ohio, United States of America; 3 Department of Genetics and Developmental Biology, Key Laboratory of Molecular Biophysics of Ministry of Education, Huazhong University of Science & Technology School of Life Science & Technology, Wuhan, Hubei, China; Aarhus University, Denmark

## Abstract

Previous studies have shown that the electroneutral Na^+^/HCO_3_^−^ cotransporter NBCn2 (SLC4A10) is predominantly expressed in the central nervous system (CNS). The physiological and pathological significances of NBCn2 have been well recognized. However, little is known about the tissue specificity of expression of different NBCn2 variants. Moreover, little is known about the expression of NBCn2 proteins in systems other than CNS. Here, we identified a set of novel *Slc4a10* variants differing from the originally described ones by containing a distinct 5′ untranslated region encoding a new extreme amino-terminus (Nt). Electrophysiology measurements showed that both NBCn2 variants with alternative Nt contain typical electroneutral Na^+^-coupled HCO_3_^−^ transport activity in *Xenopus* oocytes. Luciferase reporter assay showed that *Slc4a10* contains two alternative promoters responsible for expression of the two types of NBCn2 with distinct extreme Nt. Western blotting showed that NBCn2 proteins with the original Nt are primarily expressed in CNS, whereas those with the novel Nt are predominantly expressed in the kidney and to a lesser extent in the small intestine. Due to alternative splicing, the known NBCn2 variants contain two types of carboxyl-termini (CT) differing in the optional inclusion of a PDZ-binding motif. cDNA cloning showed that virtually all NBCn2 variants expressed in epithelial tissues contain, but the vast majority of those from the neural tissues lack the PDZ-binding motif. We conclude that alternative transcription and splicing of *Slc4a10* products are regulated in a tissue-specific manner. Our findings provide critical insights that will greatly influence the study of the physiology of NBCn2.

## Introduction

NBCn2 (*aka* NCBE), encoded by *SLC4A10*, is an electroneutral Na^+^-coupled HCO_3_^−^ transporter (NCBT) of the solute carrier 4 (SLC4) family. *SLC4A10* maps to 2q24.2 in human genome [1]. Previous studies have well established the physiological and pathological significance of NBCn2 in the central nervous system (CNS). In human, genetic disruptions in *SLC4A10* are associated with complex epilepsy, mental retardation as well as autism spectra [2]–[4]. Unusually, knockout of *Slc4a10* causes an increase in the seizure threshold in mice [5], an observation that, taken at face value, appears to be in conflict with the above reports in humans. The reason for this apparent inconsistency remains mystic. Two subsequent studies with the *Slc4a10*-null mice have shown that loss of NBCn2 expression in mice alters the protein abundance as well as the cellular targeting of a series of membrane transporters/channels as well as cytoskeleton components in the choroid plexus [6], [7]. In addition, the protein abundance of NBCn2—as well as NBCn1 (Slc4a7) and NDCBE (Slc4a8) is greatly reduced in the brains of mice subjected to chronic continuous hypoxia, suggesting that these electroneutral NCBTs are involved in the adaptive response to low-oxygen stress in CNS [8], [9].

Consistent with the significance of NBCn2 in CNS, the brain is the primary organ for the expression of NBCn2, as evidenced by northern blotting studies [10], [11]. Western blotting and immunocytochemistry studies have shown that NBCn2 is widely distributed throughout the brain [5], [12], [13]. NBCn2 is expressed in cultured and freshly dissociated hippocampal neurons [12], neurons from diverse regions of brain sections [5], [13], as well as bipolar cells and amacrine cells in the retina [14]. Although NBCn2 transcripts have been detected by RT-PCR from rat cultured astrocytes [15], NBCn2 protein is not detectable in freshly-dissociated hippocampal astrocytes [12], or those from mouse brain sections or primary cell cultures [5]. Taken together, it appears that NBCn2 is predominantly expressed in neurons, but not in high levels in astrocytes in CNS.

In addition to neurons, the choroid plexus is the second major site for the expression of NBCn2 in the brain. Here, NBCn2 is localized at the basolateral membrane of the epithelial cells [5], [7], [12], [13], [16] and likely plays an important role in the basolateral HCO_3_^−^ uptake by the choroid plexus (for review, see ref. [17]). Lack of NBCn2 in mice causes a substantial decrease in the size of the brain ventricles [5]. The above observations suggest that NBCn2 play a critical role in the cerebrospinal fluid production in mouse brain, either directly or indirectly given the aberrant expressions of a number of other proteins in NBCn2-null mice [6], [7].

*SLC4A10* contains two cassette exons that can be alternatively spliced in or out: (1) insert A encoding a 30 amino-acid (aa) cassette in the amino terminus (Nt) of NBCn2; (2) insert B which is 39 nucleotides (nt) in length and contains a stop codon [15] (see review [18]). Splicing-in insert B results in expression of a short carboxyl terminus (Ct) of NBCn2 ending with “SSPS”, whereas skipping insert B causes expression of a long Ct. This long Ct ends with “ETCL”, a typical PDZ-binding motif that can be recognized by scaffold proteins containing PDZ domains, a structure that plays critical roles in mediating the interaction between membrane proteins and cytoskeleton [19]. Hereinafter, the long NBCn2 Ct is designated as PDZ-Ct, and the alternative short Ct is designated as non-PDZ-Ct. Thus far, four major NBCn2 variants have been identified, namely, NBCn2-A through -D [20]. In addition to the two major variations of inserts A and B, a third minor variation exists in *SLC4A10* transcripts, i.e., the presence/absence of Ala^256^ due to a 3 nt shift in the splicing acceptor at the 3′-end of intron 6–7 [20]. Finally, reported as supplemental materials, a fourth variation, i.e., a small-sized insert B has been identified from human *SLC4A10* transcripts [11].

Although the physiological and pathological significance of NBCn2 have been well acknowledged, several major issues remain uncertain regarding the molecular physiology of the transporter:

1. The nature of the ion transport by SLC4A10 remains controversial. Based upon the studies with mouse (m) “NBCn2-A” [10], [21] and rat (r) “NBCn2-C” [21], two groups have proposed that NBCn2 mediates Na^+^-driven Cl^−^-HCO_3_^−^ exchange. On the other hand, Parker et al [11], based upon a study with human (h) “NBCn2-C”, have shown that hNBCn2 mediates Cl^−^-independent Na^+^/HCO_3_^−^ cotransport. They found that the Cl^−^-flux via NBCn2 is due to a Cl^−^/Cl^−^ self exchange activity of the transporter. Therefore, Parker at al. proposed to rename the transporter as “NBCn2”.
2. The physiological relevance of the structural variations in *SLC4A10* products largely remains unclear.
3. It is not clear whether *SLC4A10* contains alternative promoters.
4. It remains to be addressed whether the expression of NBCn2 variants is tissue specific.
5. Little is known about the protein expression as well as the physiological roles of NBCn2 in tissues other than CNS.

In the present study, in our attempt to address the last three issues, we made the following major findings:

1. We identified a novel exon of *Slc4a10*. Moreover, we cloned from rat and mouse a set of novel NBCn2 variants, the extreme Nt of which differs from that of the originally described NBCn2 variants. NBCn2 variants with the original Nt start with a “MEIK” motif (the first four residues), hereinafter are designated as MEIK-NBCn2. The NBCn2 variants with the novel Nt from rat start with “MCDL”, thus are referred to as MCDL-NBCn2. Those with the novel Nt from mouse start with “MQPG”, thus are designated as MQPG-NBCn2. The mouse MQPG-NBCn2 variants are orthologs of rat MCDL-NBCn2.
2. We demonstrated for the first time that *Slc4a10* contains two alternative promoters in charge of the expression of NBCn2 variants with the different extreme Nt.
3. We found that NBCn2 proteins with different Nt exhibit distinct distribution profiles in rat tissues, indicating that the transcription using alternative promoters of *Slc4a10* is highly tissue specific.
4. We found that the expression of the two types of NBCn2 Ct (i.e., PDZ-Ct vs. non-PDZ-Ct) arising from the alternative splicing of insert B is tissue-type specific, and likely cell-type specific in the brain.
5. Functional characterization with rat NBCn2 variants shows that the HCO_3_^−^ transport activities of NBCn2 with alternative Nt are not significantly different.

## Materials and Methods

### Ethics Statement

Adult specific pathogen free (SPF) mice (C57BL/6J) were purchased from the Laboratory Animal Center at Wuhan University (Wuhan, Hubei, China). Adult SPF rats (Wistar) were purchased from the Hubei Research Center of Experimental Animals (Wuhan). The mice were housed in a standard-sized small cage, and the rats were housed in a standard-sized large cage with wood chips as bedding materials and free access to rodent chow and tap water. The animals were anaesthetized with ether (administered using an open-drop method) by inhalation in a closed container until no significant response was observed to a punctuate stimulus on the feet. The animals were then sacrificed by decapitation. Organs and tissues were immediately removed. Fine dissection of brain tissues (e.g., for choroid plexus) was performed in phosphate-buffered saline on a microscope. All tissues and organs were immediately frozen in liquid nitrogen upon removal and dissection, and then stored at −80°C until usage. All protocols for animal care and usage have been approved by the Institutional Research Ethics Committee at Huazhong University of Science and Technology. Every effort was made to minimize the number of animals used as well as their suffering. *Xenopus* oocytes were prepared following a protocol approved by the Institutional Animal Care and Use Committee at Case Western Reserve University.

### 5′-Rapid amplification of cDNA ends

Total RNA was prepared using TRIzol® Reagent (Life Technologies Corporation, Carlsbad, CA, USA) previsously described [20]. The RNA preparation was dissolved in nuclease-free H_2_O and quantified with UV spectrophotometer. Agarose gel electrophoresis was always performed to examine the integrity of the RNAs.

5′-Rapid amplification of cDNA ends (RACE) was performed with total RNA from mouse brain using 5′-Full RACE Kit (TaKaRa Biotechnology Co., Ltd., Dalian, Liaoning, China). Briefly, mRNA was decapped and ligated with the 5′-RACE Adaptor provided with the kit. cDNA was then synthesized using M-MLV (Life Technologies) with anti-sense primer (5′-AGTCTACAGTGC-3′) specific to mouse *mSlc4a10*. After an unnested PCR with PrimeSTAR® HS DNA polymerase (TaKaRa) using the 5′-RACE Outer Primer (5′-CATGGCTACATGCTGACAGCCTACTG-3′, provided with the kit) and anti-sense primer N2-GSP-Outer (5′-GGTGCTCCTCATCATCGTCCTCAGTTC-3′), a nested PCR was performed using the sense primers derived from the adapter (5′-actact*cccggg*ACAGCCTACTGATGATCAGTCGATG-3′, derived from the adaptor) and anti-sense primer N2-GSP-Inner (5′-actact*gcggccgc*TCTGTGCTTATGACCACGATGCC-3′). Restrictive sites (italicized lower case sequences, the same below) were introduced into the nested primers. The PCR product was restricted, subcloned into a vector, and then transformed into bacteria. Plasmid DNA was isolated from single colonies for DNA sequencing.

### Cloning of full-length cDNA of NBCn2

Single stranded complementary DNA (cDNA) was synthesized with Moloney Murine Leukemia Virus (M-MLV) reverse transcriptase (Life Technologies). The full-length cDNA encoding NBCn2 was amplified from mouse RNA by nested reverse transcription polymerase chain reaction (RT-PCR). For the cloning of mouse (m) MEIK-NBCn2, a RT-PCR was performed using sense primer GSP-mN2-F1 (5′-CGAATACTAAGCAGAGCGAGTGCCCG-3′) and anti-sense primer GSP-mN2-R1 (5′-GAGGCTGAACACAGAAATAGAATGAAGGCTTGC-3′) followed by a nested PCR using sense primer GSP-mN2-F2 (5′-actact*cccggg*CTGAGTGGAAGACACTGAAGACACTGC-3′) and anti-sense GSP-mN2-R2 (5′-actact*gcggccgc*GTATGAAGGTGGGATGGGAGAGAGGG-3′). Similarly, for the cloning of mouse MQPG-NBCn2, an unnested RT-PCR was performed using sense primer GSP-mN2-F3 (TGCACAGAGGGGATGATGAGCAGTG) and anti-sense primer GSP-N2-R1 followed by a nested PCR using sense GSP-mN2-F4 (5′-actact*cccggg*ATGCCGAGAGACAGACATGGCTGAGC-3′) and anti-sense primer GSP-mN2-R2.

For the cloning of the full-length cDNA encoding rat (r) MCDL-NBCn2, an unnseted RT-PCR was performed with GSP-rN2-F1 (GGATGATGCACAGTGCTTGGGATACG)and GSP-rN2-R1 (GGTGTTGACCTGCTCAGAGGCTGAAC) followed by a nested PCR with GSP-rN2-F2 (atgcat*cccggg*CTGTAGATGCTGAGAGACAGAGACG) and GSP-rN2-R2 (atgcat*gcggccgc*GGATGGGAGACAGGGCTTACAATGAC).

The PCR products were digested by restrictive enzyme, subcloned into a vector, and transformed into bacteria. Plasmids were isolated from single colonies for the identification of NBCn2 variants and DNA sequencing.

### Quantitative PCR

The relative abundance of the transcripts in mouse brain encoding the two types of NBCn2 Nt was analyzed by quantitative PCR with an ABI 7900HT Fast Real-Time PCR System (Life Technologies) using Platinum® Quantitative PCR SuperMix-UDG with ROX (Life Technologies). Primers used for quantitative PCR were as follows: sense primer for mouse (m) MEIK-NBCn2: 5′-CATGGAGATTAAAGACCAGGGAG-3′, sense primer for mMQPG-NBCn2: 5′-GACAGGAAGTGTGTGATCTTTTAGG-3′, anti-sense primer for both mMEIK- and mMQPG-NBCn2: 5′-GTGTTTTGAGAATAGAGCGTGTTC-3′. TaqMan probe (FAM-CTATCCACAACGGCTTCTTCATCATTTCT-TAMRA (synthesized by Sangon Biotech Co., Ltd., Shanghai, China) was used for real-time detection of the PCR products. For qPCR assay, 4 ug of total RNA was used for cDNA synthesis in a 20 ul reaction mixture. In each qPCR assay, a series of cDNA dilutions were prepared and 1 ul of cDNA was used as template for each reaction. Four replicates were prepared for each dilution. H_2_O was used as template for negative control. For comparison of the relative abundance of the transcripts encoding mMEIK- and mMQPG-NBCn2 with alternative Nt, two sets of qPCR assays were simultaneously performed for mMEIK-NBCn2 and mMQPG-NBCn2 with a same series of dilutions of the same cDNA samples in each experiment. Threshold cycles (C_T_) was determined for mMEIK-NBCn2 and mMQPG-NBCn2 at each dilution. The ratio of the relative abundance of transcripts encoding mMEIK-NBCn2 to those encoding mMQPG-NBCn2 was determined using the formula 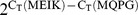 based on the C_T_ of undiluted sample.

### Fusion proteins and antibodies

The cDNA encoding the first 70 aa of mMEIK-NBCn2 Nt (a sequence that is completely identical to the counterpart of rMEIK-NBCn2 Nt) or the rat cDNA encoding the first 66 aa of rMCDL-NBCn2 Nt was amplified by PCR, and fused in frame to the 3′ end of glutathione S-transferase (GST) cDNA in pGEX-6p-1. The resulting constructs were transformed into *Escherichia coli* for expression of the fusion proteins.

Anti-MEIK polyclonal antibody has previously been described [13]. This antibody was generated against the first 18 aa (“MEIKDQGAQMEPLLPTRN”) of hMEIK-NBCn2. The sequence of this peptide is completely conserved in MEIK-NBCn2 from human, mouse, and rat. Moreover, the first 16 of the 18 aa is unique for the Nt of MEIK-NBCn2 compared to that of rMCDL-NBCn2 or mMQPG-NBCn2.

Anti-MCDL polyclonal antibody was produced by GenScript (Nanjing, Jiangsu, China) against “SGNRKVMQPGTCEHC”, a portion of the unique Nt of rMCDL-NBCn2. The last cysteine was added for conjugation to keyhole limpet hemocyanin. Anti-MCDL antibody was affinity-purified with the immunogen.

Goat-anti-rabbit secondary antibody conjugated with horse radish peroxidase was purchased from Beyotime (Haimen, Jiangsu, China).

### Membrane protein preparation and western blotting

Tissues were placed in a protein isolation buffer (in mM: 7.5 NaH_2_PO_4_, 250 sucrose, 5 EDTA, 5 EGTA, pH 7.0) containing 1% protease inhibitor cocktail for mammalian tissues (Sigma-Aldrich, St. Louis, MO, USA) and homogenized on a DY89-II homogenizer (Ningbo Scientz Biotechnology Co Ltd., Ningbo, Zhejiang, China). The lysate was then centrifuged at 3000 *g* for 15 min at 4°C to remove tissue debris. The supernatant was saved and ultracentrifuged at 10,000 *g* for 1 hr at 4°C. The pellet was then collected and dissolved in protein suspension buffer (in mM: 20 Tris-HCl, 5 EDTA, pH 8.0) containing 2% SDS. The protein concentration was determined by using Enhanced BCA Protein Assay Kit (Beyotime) according to the manufacturer's instructions. The preparation of membrane proteins was stored in aliquots at −80°C until usage.

For western blotting, membrane proteins were separated on 8% SDS-polyacrylamide gel electrophoresis (SDS-PAGE), and then transferred onto a PVDF membrane (Millipore, Bedford, MA, USA). The blot was blocked in TBST (1 mM Tris-HCl, 150 mM NaCl, 0.1% Tween20, pH 7.4) containing 5% milk for 1 hr at room temperature (RT), incubated with primary antibody at RT for 2 hr, and washed for five times with TBST. The blot was then incubated with HRP-conjugated secondary antibody (at a dilution of 1∶10,000) at RT for 1 hr, and then washed for five times with TBST. Chemiluminescence was performed with BeyoECL Plus (Beyotime) and detected with X-ray film.

### Deglycosylation

Deglycosylation was performed with Glycopeptidase F (*aka* Peptide:N-glycosidase F, TaKaRa) according to the manufacturer's instructions. Briefly, about 25 µg of membrane proteins was mixed with denature buffer supplied with the enzyme and incubated at 100°C for 3 min. The sample was then added with 1 mU of Glycopeptidase F and incubated at 37°C for 15 hr. Following the addition of 4× SDS sample buffer, the proteins were then separated on 8% SDS-PAGE for western blotting.

### Construction of expression vector for *Xenopus* oocytes, preparation and injection of cRNAs

The cDNAs encoding rNBCn2 variants were subcloned into pGH19, a *Xenopus* oocyte expression vector [22]. Our starting material was pGH19-hNBCn2-B-EGFP containing the cDNA encoding hNBCn2-B and enhanced green fluorescence protein [12]. The open reading frame of rNBCn2 was amplified by PCR, restricted with XmaI and AgeI, and used to replace the cDNA encoding hNBCn2-B on pGH19-hNBCn2-B-EGFP. The resulting constructs express rat NBCn2 variants tagged at Ct with EGFP.

For cRNA preparation, the constructs were linearized by restriction digest with NotI. cRNA was prepared using the T7 mMessage mMachine kit (Ambion, Austin, TX, USA) according to the manufacturer's instructions. Each oocyte of stage V–VI was injected with 25 ng cRNA and incubated at 18°C for 4–5 days for protein expression until being used for electrophysiology measurement.

### Electrophysiology measurements

Membrane potential (*Vm*) and intracellular pH (pHi) of an oocyte were simultaneously measured as previously described [23]. Briefly, the oocyte placed in a perfusing chamber was impaled with a proton-sensitive microelectrode for pH_i_ measurement and an electrode filled with 3 M KCl for *Vm* measurement. A third bath electrode filled with KCl was placed close to the oocyte in the chamber as reference. The signal of the electrodes was recorded using an FD223 dual-channel electrometer (World Precision Instruments, Inc., Sarasota, FL, USA) and an OC-725C oocyte clamp (Warner Instrument Corp., Hamden, CT, USA). Data were sampled every 500 ms. The following solutions were used for electrophysiology recordings.

1. ND96. This solution was nominally “HCO_3_^−^-free” and consisted of (in mM) 96 NaCl, 2 KCl, 1 MgCl_2_, 1.8 CaCl_2_, and 5 HEPES, pH 7.50, 200 mOsm.
2. 1.5% CO_2_/10 mM HCO_3_^−^. 86 (in mM) NaCl, 2 KCl, 1 MgCl_2_, 1.8 CaCl_2_, and 5 HEPES were dissolved in H_2_O, and pH was adjusted to 7.50. After addition of 10 mM NaHCO_3_, the solution was then bubbled with 1.5% CO_2_ balanced with O_2_.
3. Na-free 1.5% CO_2_/10 mM HCO_3_^−^. 86 (in mM) N-methyl-d-glucamine, 2 KCl, 1 MgCl_2_, 1.8 CaCl_2_, and 5 HEPES were dissolved in H_2_O and titrated to pH 7.50 with HCl. An additional 10 mM NMDG was added. The solution was then bubbled with 1.5% CO_2_ balanced with O_2._

### Luciferase reporter assay

Genomic DNA was extracted from mouse tissue using Ezup Column Animal Genome DNA Extracting Kit (Sangon) following the manufacturer's instructions. The genomic DNA sequence of mouse *Slc4a10* was amplified with PrimeSTAR® HS DNA polymerase (TaKaRa) and then subcloned into pGL3 vector containing a firefly luciferase gene to generate the target constructs for transcription activity analysis. The luciferase reporter assay was performed with Dual-Luciferase® Reporter Assay Systems (Progema Corporation, Madison, WI, USA) according to the manufacturer's instructions. HEK293 cells were grown to 90∼95% confluence in 96-well plates prior to plasmid transfection. A total amount of 200 ng of the target construct expressing firefly luciferase and the plasmid expressing renilla luciferase were mixed (at a mass ratio of 100∶1) with 0.5 µL Lipofectamine™ 2000 (Life Technologies) in 50 µL Opti-MEM® reduced serum media (Life Technologies) and added into each well of cells. The cells were then cultured for 24 hr, rinsed with PBS, and then incubated for 15 min at RT with 20 µL of lysis buffer provided with the Dual-Luciferase® Reporter Assay Systems (Progema). 10 µL of cell lysate was then transferred into a fresh tube, added with 20 µL of LARII and 20 µL of Stop&Glo® Reagent. Fluorescence was measured on a GLOMAX® 20/20 Luminometer (Promega). The ratio of the fluorescence signal of firefly to that of renilla was used as an index of transcription activity.

### Data analysis

Luciferase activity data as well as dpH_i_/dt data are presented as means±SE. For statistical analysis, a one-way ANOVA followed by Tukey's post hoc comparisons was performed with Minitab® 16 (Minitab Inc., State College, PA, USA). p<0.05 was considered statistically significant.

## Results

### Cloning of novel NBCn2 variants

**Figure 1:**
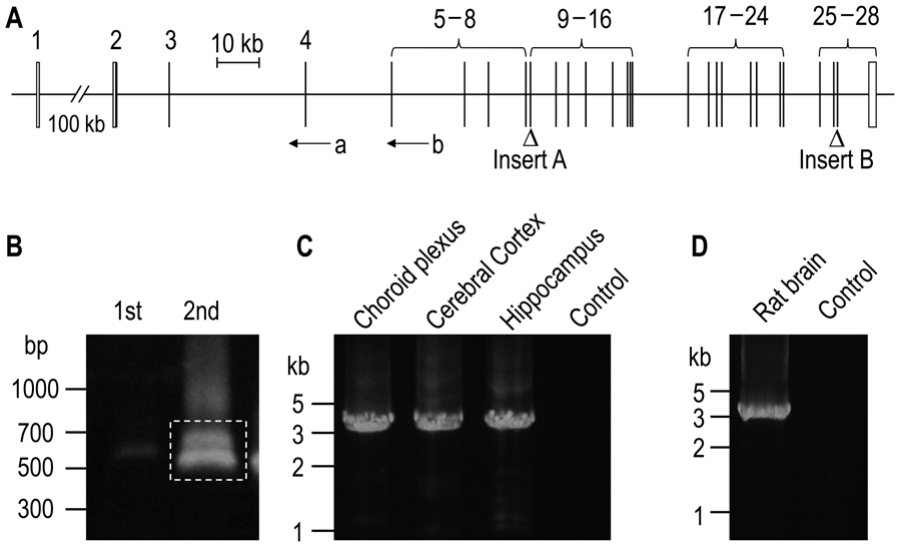
Cloning of novel NBCn2 variants. (**A**) Structure of the updated mouse *Slc4a10* gene. The updated mouse *Slc4a10* contains 28 exons (represented by vertical bars), among which the 2nd was newly identified in the present study. The white areas of the bars represent the UTRs. The exon numbers are indicated on the top of the bars. The two triangles indicate the two major cassette exons—exon 9 (insert A) and exon 27 (insert B)—that can be alternatively spliced in or out. Arrows a and b indicate the approximate positions of the anti-sense primers used for the 5′-RACE. The diagram was drawn to scale (scale bar: 10 kb). (**B**) Amplification of 5′-UTR of *Slc4a10* transcripts by nested 5′-RACE from mouse brain. The 1st lane represents the product of unnested RACE reaction, whereas the 2nd lane represents the nested PCR products. (**C**) Cloning of the full-length cDNA encoding NBCn2 variants containing the novel Nt from mouse brain tissues. (**D**) Cloning of the full-length cDNA encoding NBCn2 variants containing the novel Nt from the whole rat brain tissues. Nested RT-PCR was performed to amplify the full-length cDNA. H_2_O was used as the template for control.

By 5′-rapid amplification of cDNA ends (5′-RACE) with mouse tissues, we identified a new exon of *Slc4a10*. This new exon is predicted to encode a novel mouse (m) NBCn2 Nt starting with “MQPG”, the extremity of which is distinct from that of the previously reported mMEIK-NBCn2, namely, mNBCn2-A, -B, -C, and -D. Figure 1A shows the updated structure of mouse *Slc4a10*. Exon 1 encoding the Nt of mMEIK-NBCn2 maps to contig NT_039207.7 from nt# 2925582 to 2926051 in mouse genome, whereas exon 2 encoding the novel Nt of mNBCn2 maps to contig NT_039207.7 from nt# 3027100 to 3027490.

Figure 1B shows the two bands obtained by 5′-RACE with mouse brain tissues. Two different types of transcripts were identified from a total of 18 colonies: 16 of which represent exons 1+3+4 encoding the Nt of mMEIK-NBCn2, whereas the rest 2 clones represent exons 2+3+4 encoding the novel Nt of mMQPG-NBCn2.

The 5′-RACE data indicate that *Slc4a10* is able to express two types of transcripts that have distinct 5′ untranslated region (5′-UTR). By nested RT-PCR with sense primers specific to exon 2 of mouse *Slc4a10* and anti-sense primers complimentary to the 3′-UTR of mMEIK-NBCn2 variants, a product of ∼3.5 kb was obtained from diverse regions of mouse brain (Figure 1C). We identified from mouse brain six major novel mMQPG-NBCn2 variants. They are named as mNBCn2-E (accession #JF500487), mNBCn2-F (#JF500488), mNBCn2-G (#JF500489), mNBCn2-H (#JF500490), mNBCn2-I (#JF500487), and mNBCn2-J (#JX220977). The first four are correspondingly identical to mNBCn2-A through -D except for the extreme Nt. The last two plus a seventh clone contain an additional variation in the splicing of insert B (see details in “Identification of two additional variations of insert B”).

Using a homologous cloning approach, we obtained a product of ∼3.5 kb from rat brain by nested RT-PCR (Figure 1D). From this PCR product, we identified four rMCDL-NBCn2 variants, the Nt of which start with “MCDL”. They are named as rNBCn2-E (accession #JX073715), rNBCn2-F (#JX073716), rNBCn2-G (#JX073717), and rNBCn2-H (#JX073718). The novel rat NBCn2 variants are the orthologs of mouse NBCn2-E, -F, -G, -H, except that the novel rat variants contain an additional extension of 13 aa residues at the extreme Nt (see “Species variations in the Nt of novel NBCn2” below).

Finally, as shown in Table 1, we identified from GenBank a series of expressed sequence tags (ESTs) from diverse species as well as full-length cDNAs—accession# AK293793.1 from human encoding hNBCn2-E and BC109483.1 encoding bovine NBCn2-F—that could express NBCn2 with Nt homologous to the rMCDL-NBCn2 and mMQPG-NBCn2. Taken together, it appears that the novel NBCn2 variants are expressed in a broad range of species.

**Table 1:** cDNAs of *SLC4A10* orthologs from different species.

NBCn2 types	Species	Accession #	Tissues
Orthologs of	*Mus musculus*	CK627696.1	whole eye
MEIK-NBCn2		BG261695.1	retina
		BP758237.1	pancreatic islet
	*Danio rerio*	BI890963.1	whole embryo
		DN899623.1	eye
		EB969710.1	testis
	*Pimephales promelas*	DT193687.1	brain
	*Gasterosteus aculeatus*	DN728905.1	brain
Orthologs of	*Homo sapiens*	DC377575.1	fetal brain
rMCDL-NBCn2		DC307229.1	cerebellum
		AK293793.1	cerebellum
	*Mus musculus*	BY128043.1	brain
		BY310419.1	stroma cell
	*Bos Taurus*	DT720192.1	hypothalamus
		BC109483.1	hypothalamus
	*Gallus gallus*	BU369128.1	brain
		CV039680.1	mixed tissues
	*Taeniopygia guttata*	FK817304.1	whole brain
	*Anolis carolinensis*	FG689293.1	embryo

Note: A series of ESTs and cDNAs from the brain of human, mouse, and rat that encode the Nt of MEIK-NBCn2 are identified in GenBank. The accession numbers of these sequences are not listed here since the expression of MEIK-NBCn2 has been well studied in these species.

### Species variations in the Nt of novel NBCn2 variants

**Figure 2:**
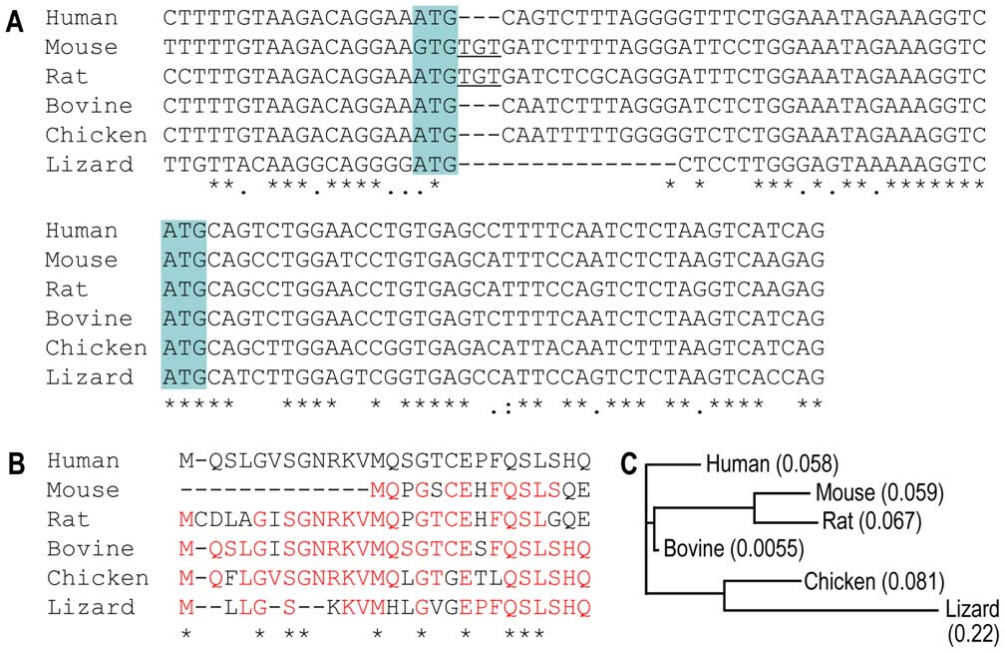
Sequence alignment of novel NBCn2 from different species. (**A**) Alignment of the 3′-portion of exon 2 of mouse *Slc4a10* and homologous exons of *SLC4A10* from other species. The two vertical gray bars indicate the two potential start codons for translation. An “A” to “G” transition mutation occurs in the first “ATG” codon in mouse *Slc4a10*. Note that shown here is the sequence derived from mouse strain C57BL/6J. An analysis on the genomic sequence (accession# AAHY01016664.1) showed that this same mutation is also present in *Slc4a10* from mouse strain 129X1/SvJ. Also note that, compared to the non-rodent species, a “TGT” insertion (underlined) occurs in mouse and rat following the first start codon. (**B**) Alignment of the predicted amino-acid sequences of the novel Nt of NBCn2 encoded by exon 2 of *SLC4A10* from different species. The alignments of DNA and amino-acid sequences were performed with multiple sequence alignment tool ClustalW2 from the European Bioinformatics Institute (http://www.ebi.ac.uk/Tools/msa/clustalw2/) followed by a manual adjustment. The asterisks “*” indicate positions of fully conserved residue. The colons “:” indicate conservation between groups of strongly similar properties, whereas the periods “.” indicate conservation between groups of weakly similar properties. (**C**) Phylogenetic tree based upon the DNA sequence alignment shown in panel A.

Interestingly, except for mouse, the sequences from other species contain two potential start codons for translation (indicated by the light blue bars in Figure 2A). In mouse, the adenine in the first start codon “ATG” is substituted by a guanine, suggesting that a transition mutation has occurred at this position in the mouse genome during its evolution. Thus, the predicted extreme Nt of the novel mouse NBCn2 variants is truncated compared to those from other species (Figure 2B). Figure 2C shows a phylogenetic analysis based on the sequence alignment in Figure 2A.

**Figure 3:**
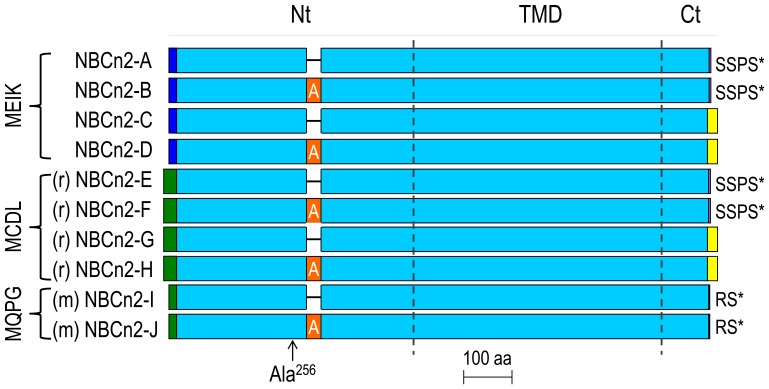
Diagram of primary structures of NBCn2 variants. The unique portion (16 aa) of the MEIK-NBCn2 Nt are represented in blue. The unique Nt of novel rat and mouse NBCn2 variants are represented in green. Compared to mouse, the rat novel Nt contains an additional extension of 13 aa residues (see Figure 2B). Insert A (30 aa, orange) is encoded by exon 9 in Figure 1A. The unique long Cts (21 aa, indicated by yellow) of NBCn2-C, -D, -G, and -H contain a PDZ-binding motif “ETCL”. The unique sequences of the two non-PDZ-Ct are indicated at the right end. Ala^256^ (position 256 relative to the first Met of MEIK-NBCn2) may be omitted in MEIK-NBCn2 [25] as well as the novel NBCn2 variants identified in the present study (i.e., rat NBCn2-F and rat NBCn2-H). The diagram was drawn to scale (scale bar: 100 aa). The sequence alignment was based on human NBCn2-A (accession# NP_071341), human NBCn2-B (#AAQ83632), rat NBCn2-C (#AAO59639), mouse NBCn2-D (#ADX99207), rat NBCn2-E (#AFP48940), rat NBCn2-F (#AFP48941), rat NBCn2-G (#AFP48942), rat NBCn2-H (#AFP48943), mouse NBCn2-I (#AFQ60533), mouse NBCn2-J(#AFN27376).

Figure 3 shows the diagram of all known major NBCn2 variants. In summary, these NBCn2 variants comprise of three major variations—alternative Nts, optional inclusion of insert A, and alternative Cts. In addition, the NBCn2 variants contain a minor variation, i.e., the optional inclusion of a single Ala residue (position Ala^256^ in MEIK-NBCn2).

### Identification of two additional variations of insert B

**Figure 4:**
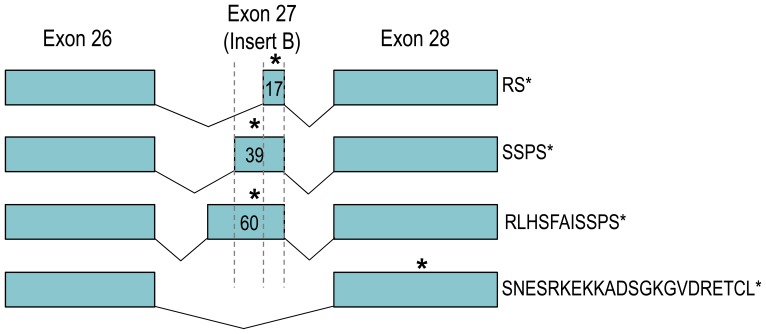
Variations and alternative splicing of exon 27 (insert B). Originally described to contain 39 nt [15], the new insert B contains 60 nt and can be spliced by four different mechanisms, leading to the expression of four different NBCn2 Ct. The diagram shows the exon structures encoding the four types of NBCn2 Ct. The unique Ct amino-acid sequences are shown at the right end of each transcript. Insert B contains two stop codons (indicated by stars). Splicing-in of the 17-nt insert B leads to expression of the short “RS” Ct using the second stop codon. Splicing-in of either the 39-nt or the 60-nt insert B leads to the expression—using the first stop codon—of two NBCn2 Ct, both ending with an “SSPS” motif but differing in the presence/absence of a 7-aa cassette “RLHSFAI”. Finally, skipping of the entire exon extends the open reading frame to exon 28, resulting in the expression of the longest NBCn2 Ct containing a PDZ-binding motif “ETCL”. The numbers in the boxes indicate the length of insert B.

The alternative splicing of insert B (exon 27) was originally described by Giffard et al. [15]. This alternative unit originally has 39 nt containing a stop codon. As shown in Figure 4, skipping insert B causes expression of the long Ct containing a PDZ-binding motif “ETCL”. Inclusion of the 39-nt insert B leads to expression of a short non-PDZ-Ct ending with a “SSPS” motif, thus lacking the PDZ-binding motif.

Here, we identified from mouse brain two additional variations in the splicing of insert B: one small-sized, and the other large-sized. As shown in Figure 4, the small-sized insert B contains only the last 17 nt of exon 27. Inclusion of this small insert B leads to expression of a second non-PDZ-Ct with “SSPS” replaced by “RS”. We identified two such variants containing the small insert B: mNBCn2-I from olfactory bulb (OB) and mNBCn2-J from cerebral cortex (CX). Note that, the small insert B expressed in mice is similar to that in a partial human cDNA clone [11].

The large-sized insert B contains 60 nt due to an extension at the 5′ end of exon 27. Inclusion of this large-sized insert B leads to expression of a third non-PDZ-Ct containing a cassette (“RLHSFAI”) before the “SSPS” motif. Unusually, a non-canonical splicing site pair “GT···AT” is used for the leading intron of the large insert B. We identified only one such variant (accession #JX268544) which is identical to mNBCn2-F except for the size of insert B. Thus, at this stage, we did not propose to assign a unique letter for this variant.

### HCO_3_^−^ transport activities of NBCn2 variants with alternative Nt

To examine the HCO_3_^−^ transport activities of NBCn2 variants with alternative Nt, we heterologously expressed rNBCn2-C, rNBCn2-G in *Xenopus* oocytes. Moreover, we created a rNBCn2-G mutant (rNBCn2-GΔNt) starting from “MQPG” to mimic the species variation in the novel mouse NBCn2 Nt. All these NBCn2 contained an EGFP tag at their Ct. For electrophysiology recordings, an oocyte was placed in a chamber and superfused with nominally “HCO_3_^−^-free” ND96 solution until *Vm* and pH_i_ were stable. The cell was then exposed to 1.5% CO_2_/10 mM HCO_3_^−^ in the presence of Na^+^, followed by a removal of extracellular Na^+^ in the continuous presence of CO_2_/HCO_3_^−^. The oocyte was subjected to a second exposure of CO_2_/HCO_3_^−^ in the presence of Na^+^ prior to returning to ND96.

**Figure 5:**
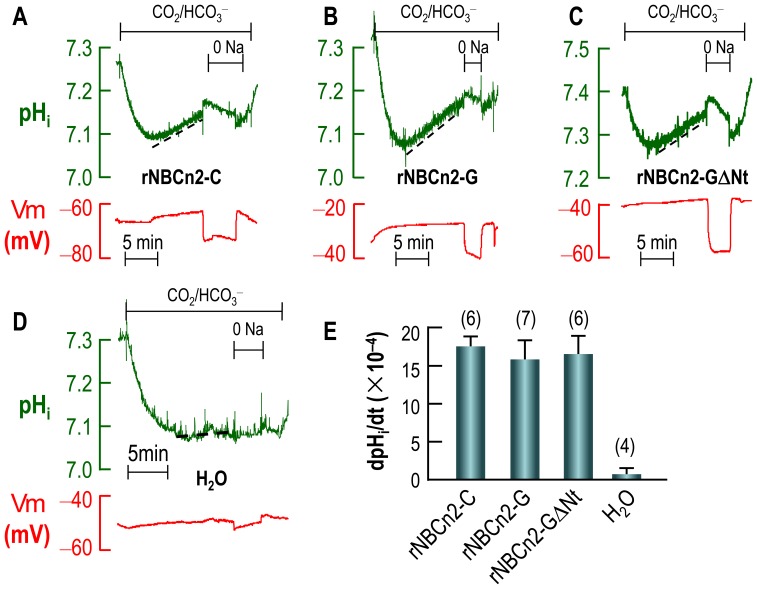
Functional characterization of NBCn2 variants. The oocytes were superfused with nominally HCO_3_^−^-free ND96. An acid load was introduced by exposing the cells to 1.5% CO_2_/10 mM HCO_3_^−^ which was followed by a removal of extracellular Na^+^. Intracellular pH and membrane potential *Vm* of the oocytes were simultaneously recorded with microelectrodes. Representative recordings of pH_i_ and *Vm* are shown for rNBCn2-C-EGFP (**A**), rNBCn2-G-EGFP (**B**), rNBCn2-GΔNt-EGFP (**C**), and H_2_O-injected control oocyte (**D**). (**E**) Summary of pH_i_ recovery rates (dpH_i_/dt) of oocytes expressing NBCn2 variants/mutant or injected with H_2_O. The dpH_i_/dt of NBCn2-expressing oocytes are all significantly different from that of H_2_O-injected control oocytes.

Figure 5A shows typical recordings of intracellular pH (pH_i_) and membrane potential (*Vm*) from an oocyte expressing rNBCn2-C. Introduction of CO_2_/HCO_3_^−^ caused a rapid fall in pH_i_ due to influx of CO_2_. The pH_i_ then gradually recovered from the CO_2_-induced acidification. The rate of pH_i_ recovery (dpH_i_/dt as represented by the slope of the dashed line) is an index of the HCO_3_^−^ transport activity of NBCn2. Similarly, the oocyte expressing rNBCn2-G (Figure 5B) or rNBCn2-GΔNt (Figure 5C) displayed strong pH_i_ recovery upon the CO_2_-induced acidification. In contrast, the control oocyte injected with H_2_O showed no significant pH_i_ recovery (Figure 5D). The mean dpH_i_/dt of oocytes expressing rNBCn2-C, rNBCn2-G, or rNBCn2-GΔNt are not significantly different from each other (Figure 5E).

Consistent with the electroneutral transport nature of NBCn2, no abrupt changes in *Vm* was observed upon the introduction of CO_2_/HCO_3_^−^ for any of these NBCn2. However, the removal of extracellular Na^+^ caused a modest hyperpolarization, presumably due to an Na^+^ leakage. This Na^+^ leakage could be mediated by NBCn2, as is the case for NBCn1 which contains an intrinsic ion conductance [24]. It could also be mediated by an endogenous Na^+^-conductance in *Xenopus* oocyte that was activated by the presence of NBCn2.

### Characterization of *Slc4a10* promoters

The expression of two types of transcripts with distinct 5′-UTR suggests that *SLC4A10* contain two promoters in charge of expression of two types of NBCn2 with alternative Nt. To test this hypothesis, we amplified the genomic sequences of mouse *Slc4a10* and performed luciferase reporter assay.

**Figure 6:**
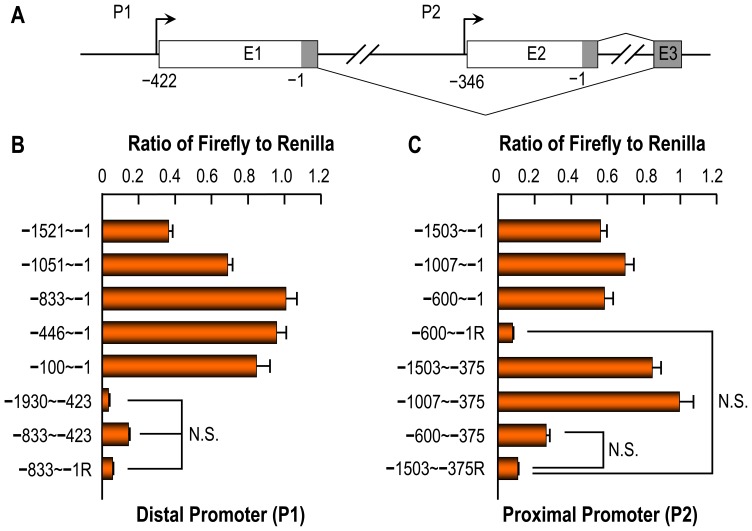
Characterization of mouse *Slc4a10* promoters. (**A**) Diagram of the minimal promoter regions of mouse *Slc4a10*. *Slc4a10* contains two alternative promoters: the distal P1 and the proximal P2. Shown here are only the first three exons (E1–E3) of *Slc4a10*. The gray areas indicate the coding regions of the exons. The numbers indicate the nucleotide positions relative to the start codon “ATG”, with “−1” representing the first nucleotide upstream of the start codon encoding “MEIK” or “MQPG” of mouse NBCn2. The arrows indicate the transcription initiation site. The connections between the exons indicate the splicing of *Slc4a10* transcripts produced from the alternative promoters. Exon 2 is omitted in the mature *Slc4a10* transcripts produced from P1. (**B**) Characterization of the distal promoter (P1) expressing mMEIK-NBCn2. (**C**) Characterization of the proximal promoter (P2) expressing mMQPG-NBCn2. For transcription activity assay, the genomic sequence was subcloned into pGL3 vector containing firefly luciferase reporter gene. The constructs with “R” indicate that the corresponding sequences were subcloned into pGL3 in reverse direction. A plasmid containing the renilla luciferase gene was simultaneously transfected with pGL3 containing the firefly luciferase gene. The ratios of the fluorescence intensity of firefly to that of renilla were used as indices of the transcription activities. Each bar represents the mean of at least three independent experiments. Quadruplicates were prepared for each construct in each experiment.

Figure 6A shows the structure of promoter regions of mouse *Slc4a10*. As summarized in Figure 6B, for the distal promoter P1 expressing mMEIK-NBCn2, all constructs containing the sequence to −1, thus including the 5′-UTR, elicited transcription activities in HEK cells that are substantially higher than the control construct “−833∼−1R” containing the sequence from −833 to −1 in reverse direction. Interestingly, compared to construct “−833∼−1”, the construct containing just the sequence from −100 to −1 virtually retained the full transcription activity. Note that, removal of the 5′-UTR (constructs “−1930∼−423” and “−833∼−423”) largely abolished the transcription activity, indicating that the 5′-UTR is essential for the efficient transcription from the distal promoter P1.

For the proximal promoter P2 expressing mMQPG-NBCn2 (Figure 6C), all three constructs containing a sequence to −1 elicited substantial transcription activities compared to the control construct “−600∼−1R” containing the sequence from −600 to −1 oriented in reverse direction. Unlike the distal promoter p1, both “−1053∼−375” and “−1007∼−375”, both with the 5′-UTR removed, retained substantial transcription activities compared to the control construct “−1503∼−375R” containing the sequence from −1503 to −375 placed in reverse direction. Construct “−600∼−375” lacking both 5′-UTR and the sequence upstream of −600 only exhibited very basal transcription activity (compared to control “−1053∼−375R”: p = 8.7×10^−8^ by two-tailed student's T-test, not significantly different by ANOVA). Interestingly, except for construct “−600∼−375”, those lacking the 5′-UTR exhibited slightly higher transcription activities than the corresponding ones containing the 5′-UTR (“−1503∼−1” vs. “−1503∼−375”, “−1007∼−1” vs. “−1007∼−375”, significantly different by ANOVA). The results indicate that, unlike the distal promoter P1, two separated elements of promoter P2, i.e., the 5′-UTR and the sequence from −1007 to −600, can evoke substantial transcription activity. However, it is likely that some kind of interaction—the nature of which remains to be elucidated—exist between the 5′-UTR and the element from −1007 to −600, as evidenced by the decreased transcription efficiency when these two elements were simultaneously present.

### Tissue specific expression of NBCn2 with alternative Nt

**Figure 7:**
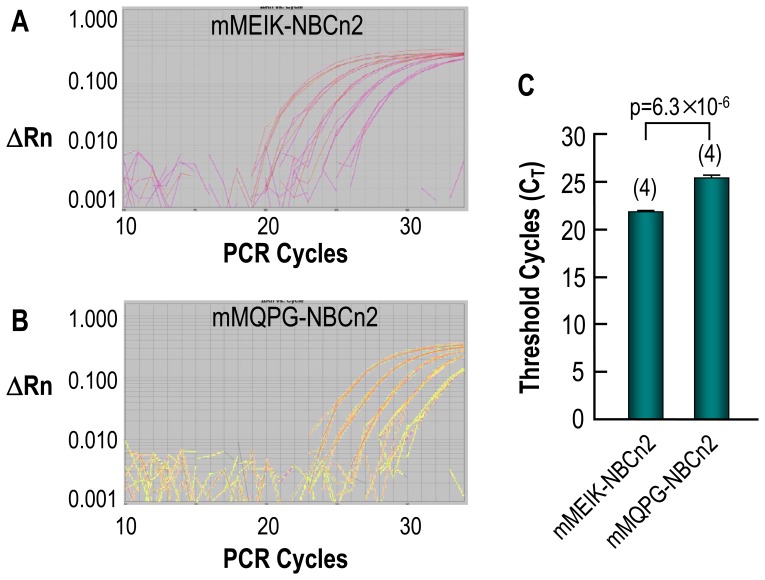
Quantitative PCR analysis of the relative abundances of transcripts encoding mMEIK-NBCn2 and mMQPG-NBCn2 in mouse brain. (**A**) Representative amplification plot of qPCR for the transcripts encoding the total of mMEIK-NBCn2 in mouse brain. (**B**) Representative amplification plot of qPCR for the transcripts encoding the total of mMQPG-NBCn2 in mouse brain. (**C**) Summary of threshold cycles (C_T_) for mMEIK-NBCn2 and mMQPG-NBCn2. The amplification efficiencies were 91.7±2.4% (n = 5) for mMEIK-NBCn2, and 93.1±3.5% (n = 5) for mMQPG-NBCn2, p = 0.76 by student's t-test. The data in panel C represent the summarized C_T_ for the undiluted cDNA samples obtained from four experiments like those shown in panels A and B. ΔRn: normalized reporter signal of qPCR products subtracted by the background signal. Two-tailed student's t-test was performed for statistical comparison between the amplification efficiencies and C_T_ values of mMEIK- vs. mMQPG-NBCn2.

Given that, as shown by previous studies (see Introduction), NBCn2 transcripts are predominantly expressed in the brain, we performed quantitative PCR (qPCR) with mouse brain to examine the relative abundance of the transcripts for mNBCn2 with the original Nt vs. those for mNBCn2 with the novel Nt. Representative amplification plots were shown for mMEIK-NBCn2 (Figure 7A) and mMQPG-NBCn2 (Figure 7B). The threshold cycles (C_T_) were 21.8±0.1 (n = 4) for mMEIK-NBCn2 vs. 25.4±0.2 (n = 4) for mMQPG-NBCn2, p = 6.3×10^−6^. The ratio of the abundance of the transcripts encoding mMEIK-NBCn2 to that of the transcripts encoding mMQPG-NBCn2 was 12 (2^25.4-21.8^≈12).

The qPCR data are somewhat consistent with our aforementioned 5′-RACE data, which showed that the frequency of the colonies representing mMEIK-NBCn2 was 8 times higher than that of the colonies representing mMQPG-NBCn2. Together, the data indicate that the usage of the distal promoter is by far dominant for the expression of *Slc4a10* in mouse brain.

**Figure 8:**
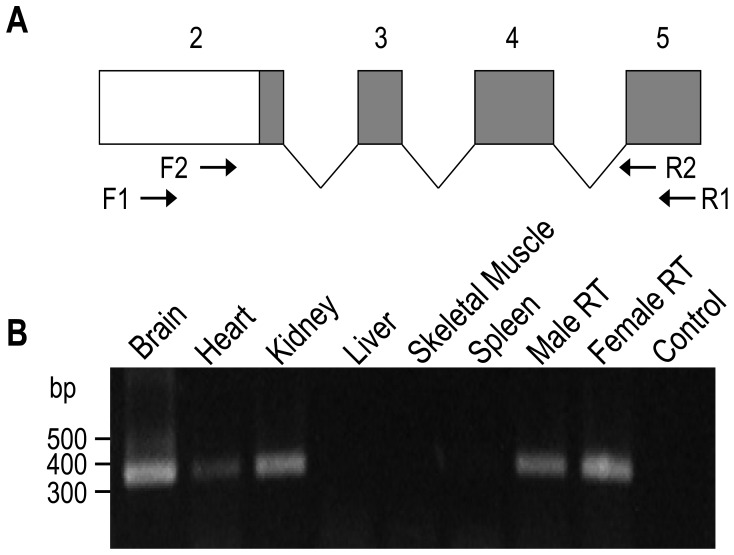
Expression of transcripts encoding novel NBCn2 variants in mouse tissues. (**A**) Diagram to show the first four exons of the transcripts encoding the novel NBCn2 variants. Exon numbers (consistent with the numbering in Figure 1A) are marked on the top. The open area of the bar indicates the non-coding region of exon 2, whereas the gray areas represent the coding regions. The arrows indicate the approximate locations of the two sets of primers used for the nested RT-PCR. (**B**) RT-PCR analysis of transcripts encoding the novel NBCn2 variants in mouse tissues. By nested RT-PCR, a product of ∼340 bp was obtained from the brain, heart, kidney, male as well as female reproductive tract (RT) tissues. The male RT represented the mixture of testis and epididymis. The female RT represented the mixture of ovary, uterus, and vagina. H_2_O was used as template for control.

By RT-PCR, we then examined the expression of transcripts encoding mMQPG-NBCn2 in more selected mouse tissues (Figure 8). The results showed that alternative transcripts encoding mMQPG-NBCn2 derived from the proximal promoter are expressed in the brain, heart, kidney, and male as well as female reproductive tract tissues.

**Figure 9:**
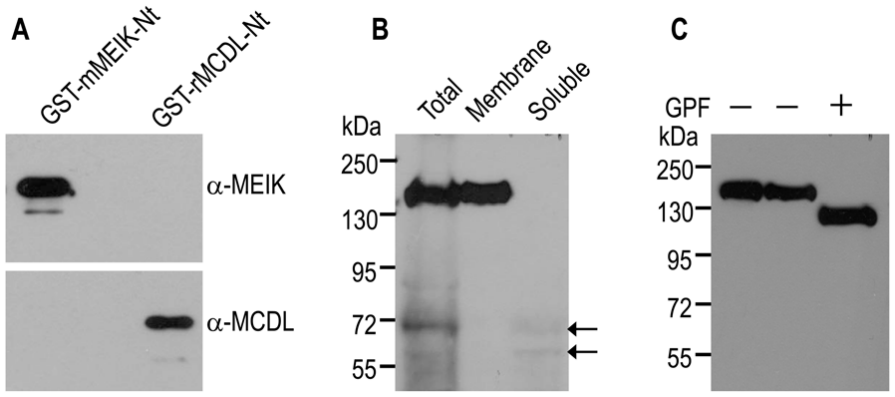
Validation of polyclonal antibodies, expression and deglycosylation of NBCn2 in kidney. (**A**) Validation of NBCn2 antibodies by western blotting with GST-fusion proteins. The whole lysate of bacteria expressing the GST-fusion proteins were separated by two parallel 10% SDS-PAGE, and transferred onto two PVDF membranes. (**B**) Validation of anti-MCDL by western blotting with the protein preparations from rat kidney. The lane “Total” represents 300 µg of whole lysate, whereas the “Membrane” and “Soluble” represent the pellet and supernatant obtained from an amount of whole lysate equivalent to that loaded in lane “Total”. (**C**) Deglycosylation of NBCn2 proteins expressed in rat kidney. The protein preparations were treated in the presence or absence of Glycopeptidase F (GPF). In the experiments for panels B and C, the proteins were separated on 8% SDS-PAGE. All antibodies were used at a dilution of 1∶5000.

We further examined in rat tissues the protein distribution of NBCn2 with different types of Nt (MEIK- vs MCDL-NBCn2). We used an anti-MEIK antibody directed against the first 18 aa of hMEIK-NBCn2 that was previously described [13]. We also developed a new polyclonal antibody anti-MCDL against the unique portion of rMCDL-NBCn2. The antibodies were validated with fusion proteins GST-mMEIK-Nt and GST-rMCDL-Nt. As shown in Figure 9A, the anti-MEIK antibody specifically recognized GST-mMEIK-Nt, but not GST-rMCDL-Nt. In contrast, our new anti-MCDL antibody specifically recognized GST-rMCDL-Nt, but not GST-mMEIK-Nt. Note that, the Nt sequence of mMEIK-NBCn2 in fusion protein GST-mMEIK-Nt is completely identical to the counterpart of the Nt of rMEIK-NBCn2. The data demonstrated that the two antibodies are able to distinguish the two types of NBCn2 variants with different extreme Nt.

To further characterize the new antibody against rMCDL-NBCn2, we performed western blotting with protein preparations from rat kidney. Anti-MCDL antibody recognized a band with molecular weight (MW) of ∼150 kDa in the crude lysate (Lane “Total” in Figure 9B) as well as membrane proteins (Lane “Membrane”), but not in the soluble proteins (Lane “Soluble”). The two lower bands (indicated by arrows) in lanes “Total” and “Soluble” presumably represent the degraded products of NBCn2. As shown in Figure 9C, deglycosylation with Glycopeptidase F (GPF) reduced the MW of NBCn2 from ∼150 to ∼125 kDa (predicted MW of rMCDL-NBCn2 ranges from 123.7 to 125.7 kDa). Taking together, we conclude that the anti-MCDL is specific and that NBCn2 proteins with the novel Nt are expressed in rat kidney.

**Figure 10:**
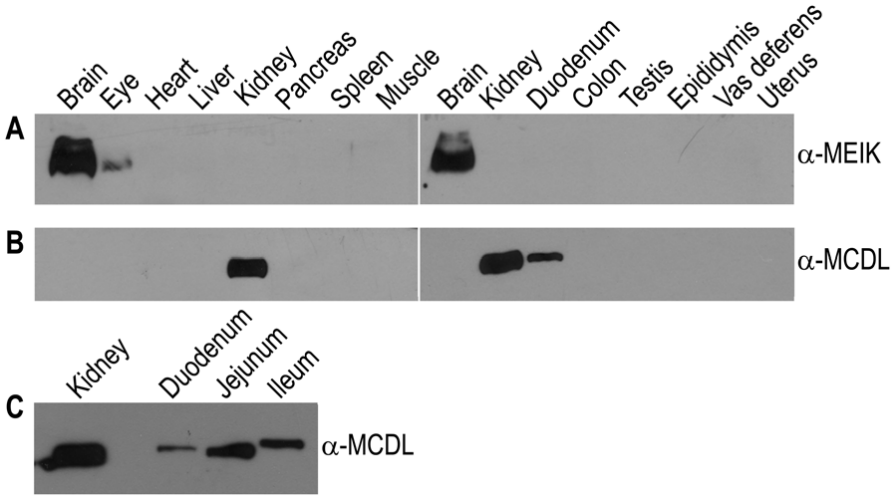
Expression of NBCn2 proteins in rat tissues. (**A**) Expression of NBCn2 with the originally described Nt in rat tissues. (**B&C**) Expression of NBCn2 with the novel Nt in rat tissues. For each tissue, equal amount (50 µg) of membrane proteins were separated on 8% SDS-PAGE, and blotted onto PVDF membrane. Two parallel blots were simultaneously prepared, and probed with anti-MEIK or anti-MCDL at a dilution of 1∶5000.

Using anti-MEIK and anti-MCDL antibodies, we studied the distribution profiles of the two types of NBCn2 proteins with alternative Nt in rat tissues. The results showed that anti-MEIK immunoreactivity is primarily distributed in the brain, in much lower level in the eyes, and not detectable in other studied tissues (Figure 10A). In contrast, anti-MCDL immunoreactivity is most abundant in the kidney, and present to a lesser extend in the duodenum, jejunum, and ileum (Figure 10B&C).

Taken together, our data demonstrated that the two types of NBCn2 with alternative Nt have distinct distribution profiles in rat tissues. In other words, the usage of the alternative promoters in the expression of *Slc4a10* is highly tissue specific.

### Tissue specific expression of NBCn2 with alternative Ct

**Figure 11:**
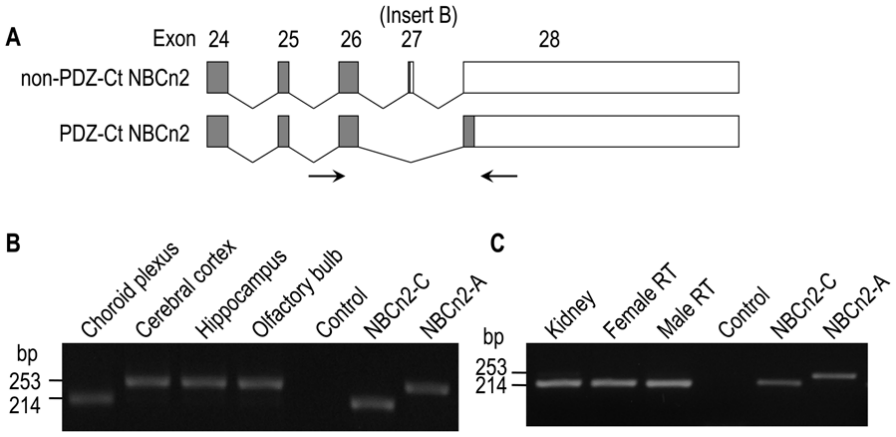
Tissue-specificity of expression of transcripts encoding mouse NBCn2 variants with alternative Ct. (**A**) Exon structures of the Ct portion of NBCn2 variants. The transcripts encoding NBCn2 with non-PDZ-Ct contain insert B (exon 27), whereas those encoding NBCn2 with PDZ-Ct lack insert B. The exon numbers (consistent with the numbering in Figure 1A) are indicated on the top of each box. The gray bars represent the coding regions, whereas the open boxes represent the UTRs. The arrows indicate the primers used in the RT-PCR analysis. (**B**) RT-PCR analysis of the expression of insert B in brain tissues. (**C**) RT-PCR analysis of the expression of insert B in the kidney, male as well as female reproductive tract (RT). The female reproductive tract included the mixture of the ovary, uterus, and vagina. The male reproductive tract included the mixture of the testis, epididymis, and vas deferens. The PCR products obtained with a plasmid containing mouse NBCn2-A (with insert B) or NBCn2-C (lacking insert B) were served as MW markers.

Due to alternative splicing of insert B (exon 27), *SLC4A10* is able to produce two major types of NBCn2 Ct (PDZ-Ct vs. non-PDZ-Ct). To examine whether the expression of insert B is tissue specific, we performed RT-PCR analysis with mouse tissues using primers flanking insert B (Figure 11A). As shown in Figure 11B&C, a DNA product of 214 bp was amplified from the choroid plexus, kidney, and male as well as female reproductive tract tissues. The data indicate that insert B is predominantly omitted in the *Slc4a10* transcripts expressed in these epithelial tissues. In contrast, a 253-bp fragment was obtained from the neural tissues including the cerebral cortex (CX), hippocampus (HC) as well as olfactory bulb (OB), indicating that insert B is predominantly included in the *Slc4a10* transcripts expressed in the neural tissues. Taken together, our data indicate that alternative splicing of insert B in *Slc4a10* transcripts is tissue-type specific in mouse.

We further examined the tissue specificity of the alternative splicing of *Slc4a10* transcripts by full-length cDNA cloning using a semi-quantitative approach [25]. The full-length cDNAs encoding mMEIK-NBCn2 or those encoding mMQPG-NBCn2 were amplified by RT-PCR, subcloned into a vector, and transformed into bacteria for identification of NBCn2 variants from colonies. Summarized in Table 2 are the frequency distribution data of different NBCn2 variants. In the central nervous system, mNBCn2-A accounts for 131/(131+31+8) = 77% in CX, 76% in HC, and 87% in OB of the total of mMEIK-NBCn2 expressed in each of these tissues. The sum of mNBCn2-A plus -B—both containing the short non-PDZ-Ct—accounts for 95% in CX, 89% in HC, and 94% in OB of the total of mMEIKNBCn2. Similarly, mNBCn2-E accounts for 55% in CX, 83% in HC, and 91% in OB of the total of mMQPG-NBCn2 expressed in each of these tissues. The sum of mNBCn2-E plus -F—both containing the non-PDZ-Ct—accounts for 69% in CX, 93% in HC, and 97% in OB of the total of mMQPG-NBCn2 expressed in each of these tissues. Taken together, mNBCn2 variants with the non-PDZ-Ct account for the vast majority of the total mNBCn2 expressed in the neural tissues of CX, HC, and OB.

**Table 2:** Colony frequency distribution of NBCn2 variants in mouse tissues.^a^

Tissue	mNBCn2 Variants									
	A	B	C	D	E	F	G	H	I	J
CX	131	31	8	0	73	17	37	4	0	1
HC	48	8	6	1	62	8^b^	5	0	0	0
OB	43	2	4	0	53	2	3	0	2	0
CP	0	0	59	2	0	0	67	2	0	0
Kidney^c^	0	0	56	6	NA	NA	NA	NA	NA	NA
RT♀	0	0	65	0	0	0	82	0	0	0
RT♂	0	0	107	0	0	1	70	0	0	0

a The colony frequency distribution was determined as described previously [20]. Take mMEIK-NBCn2 in CX as example, the cDNA encoding the total of these variants in CX was amplified by one set of RT-PCRs, subcloned into a vector, and then transformed into bacteria. The NBCn2 variant from a single colony was determined by examining the presence/absence of inserts A and B by PCR. Among the total of 170 (131+31+8 = 170) colonies identified from CX, 131 was mNBCn2-A, 31 was NBCn2-B, 8 was mNBCn2-C, and none was NBCn2-D. The colony frequency distribution of each of mMQPG-NBCn2 was determined by a similar approach using a set of PCR primers specific for mMQPG-NBCn2. CX: cerebral cortex; HC: hippocampus; OB: olfactory bulb; CP: choroid plexus; RT♀: female reproductive tract including ovary, uterus and vagina; RT♂: male reproductive tract including testis, epididymis, and vas deferens.

b 1 of the 8 clones contained the 60-nt insert B and is annotated as NBCn2-F.

c It was extremely difficult to obtain a product for mMQPG-NBCn2 from mouse kidney by RT-PCR. We were lucky to success in only one experiment, from which all 42 colonies identified were mNBCn2-G. These clones were unlikely derived from sample contamination as we processed just the mouse kidney during the whole experiment. On the other hand, by RT-PCR, it was very easy to obtain a product from rat kidney for rMCDL-NBCn2, the orthologs of mMQPG-NBCn2. Consistent with the case of mouse kidney, all variants from rat kidney were rNBCn2-G (data not shown).

In striking contrast, mNBCn2-C virtually accounts for all in the total of mMEIK-NBCn2 and mNBCn2-G virtually accounts for all in the total of mMQPG-NBCn2 identified from the choroid plexus. No mNBCn2 variant with the non-PDZ-Ct was identified from the choroid plexus. Similarly, mNBCn2-C and -G virtually account for all the mNBCn2 variants identified from the kidney and male as well as female reproductive tract tissues.

Thus, consistent with the RT-PCR data in Figure 11, the full-length cDNA cloning data in Table 2 again demonstrated that insert B is predominantly included in the studied neural tissues. In contrast, it is by far predominantly, if not exclusively, omitted in the *Slc4a10* transcripts expressed in the epithelial tissues. In summary, our data demonstrated that the expression of the PDZ-Ct vs. non-PDZ-Ct in NBCn2 caused by the alternative splicing of insert B is tissue-type specific in mouse.

Consider the alternative splicing of insert A (exon 9) which encodes a 30-aa peptide in the cytoplasmic Nt domain of NBCn2. Among all eight NBCn2 variants, NBCn2-A/C/E/G do not contain insert A (Figure 3). Note that, in all cases in Table 2, the vast majority of the mNBCn2 variants—the sum of mNBCn2-A plus -C in the total of mMEIK-NBCn2, and the sum of mNBCn2-E plus -G in the total of mMQPG-NBCn2—are those lacking insert A. The data suggest that insert A is predominantly skipped in mNBCn2 expressed in these tissues.

Back to the central nervous system, we have shown that mNBCn2-A accounts for the vast majority of the total of mMEIK-NBCn2 expressed in CX, HC, and OB. The data are consistent with a previous estimation by Liu et al showing that mNBCn2-A accounts for the vast majority of the total mMEIK-NBCn2 in mouse brain [20]. Although virtually all mNBCn2 variants are mNBCn2-C/G and no mNBCn2-A is expressed in the choroid plexus, the previous estimation by Liu et al is still valid given that the choroid plexus only accounts for a very tiny portion of the whole mass of the brain. Recall that, our qPCR data have shown that the transcripts encoding mMEIK-NBCn2 are 12 times more abundant than those encoding mMQPG-NBCn2 in the brain (Figure 7). Thus, it is reasonable to speculate that mNBCn2-A is by far dominant among all eight mNBCn2 variants expressed in the brain.

## Discussion

### Sources of structural variations in *Slc4a10* products

Alternative transcription using distinct promoters and alternative splicing are common strategies for the regulation of gene expression (for review, see refs. [26], [27]). The transcriptional (using alternative promoters) and post-transcriptional (alternative splicing) regulations greatly increase the coding capacity of the genome, thus greatly increase the diversity of expression products. Not surprisingly, all five NCBTs of the SLC4 family are able to produce multiple variants by alternative transcription using distinct promoters and/or alternative splicing of pre-mRNAs (see review [18], [28]). For example, *SLC4A4* encoding the electrogenic Na^+^/HCO_3_^−^ cotransporter NBCe1 contains two alternative promoters, capable of expressing two types of proteins differing in the Nt extremity [29]. In addition, *SLC4A4* contains two cassette exons that can be alternatively spliced in or out [20], [30]. *SLC4A5* is capable of producing multiple NBCe2 variants by alternative splicing (see review [18]). Moreover, *SLC4A5* contains at least two alternative promoters [31], although no alternative Nt of NBCe2 has been reported. Both *SLC4A7* encoding NBCn1 (see review [18]) and *SLC4A8* encoding NDCBE [32] are able to produce two types of extreme Nt presumably under the control of alternative promoters. NBCn1 contains at least three alternatively-spliced cassettes (see review [18], [28]). NDCBE contains two types of extreme Ct derived from alternative splicing of exon 22 in *SLC4A8* [32].

The known NBCn2 variants contain three major structural variations that involve the usage of alternative promoters and/or splicing in/out of an entire exon. These major structural variations include:

1. Two alternative Nts derived from distinct promoters using exons 1 or 2. The present study is the first demonstration that *Slc4a10* contains two promoters in charge of expression of the two alternative Nts of NBCn2. Note that, compared to other species, mouse *Slc4a10* contains an additional species-specific variation in exon 2, resulting in a “truncation” in the novel Nt.
2. Optional inclusion of the 90-nt insert A (exon 9) encoding the 30aa cassette.
3. Four alternative Cts derived from the alternative splicing of insert B (exon 27). Skipping the entire insert B leads to expression of the long Ct containing a PDZ-binding motif. Inclusion of the 17-nt insert B leads to the expression of a non-PDZ Ct ending with “RS” motif. Inclusion of the 39-nt insert B results in the expression of a second non-PDZ Ct ending with “SSPS”. Finally, inclusion of the 60-nt insert B results in the expression of a third non-PDZ Ct ending with “RLHSFAISSPS”.

In addition to the three major structural variations, *Slc4a10* products contains a minor variation, namely, the optional inclusion of a single alanine residue (position Ala^256^ in MEIK-NBCn2) due to alternative splicing of the three nucleotides at the 5′-end of exon 8.

Consider just the other three alternative Ct but not the non-PDZ-Ct “RLHSFAISSPS” which we have observed only in one single clone. The combination of the two alternative Nts+presence/absence of insert A+three alternative Cts can produce up to 12 major NBCn2 variants. We propose to assign a unique English letter for each identified major variant in the order of discovery. Of the 12 potential major NBCn2 variants, 10 (NBCn2-A through -J) have been identified. Of these 10 known, 6 are reported for the first time in the present study.

Note that, the above calculation does not count for the species-specific variations in the novel Nt of NBCn2, e.g., mouse “MQPG” vs rat “MCDL” vs human “MQSL” (Figure 2), because the variants with these Nt are derived from the same mechanism using the homologous alternative promoter. Thus, any such variants from different species with the same combination of other alternative structural elements (insert A and Ct) would be named with the same English letter, e.g., mouse NBCn2-E vs rat NBCn2-E.

Finally, note that, our definition of major vs minor variations is simply structural and has no implication in the functional significance of the alternative structural elements.

Our study has greatly increased the diversity of Slc410 products. The diversity of Slc4a10 products may have important physiological relevance. Firstly, different combinations of alternative structural elements may define different intrinsic HCO_3_^−^ transport activities of the transporter, as is the case for NBCe1 [33]. In the present study, we have examined the effect of the structural variation in the extreme Nt on the functional expression—which is the product of surface abundance and intrinsic HCO_3_^−^ transport activity—of rNBCn2-C vs rNBCn2-G. The results show the variation in the extreme Nt has no significant effect on the functional expression of these two specific variants in the context of *Xenopus* oocytes. A more systemic study is required to address the functional effects of the alternative structural elements on the activities of NBCn2 variants.

Secondly, NBCn2 variants with specific combinations of alternative structural elements might be expressed in specific tissues and even cell types (discussed below) or at specific developmental stages, therefore performing differential physiological roles under different physiological context.

Thirdly, different combinations of alternative structural elements may affect the functional modulation of the transporter via different signaling cascades (discussed below).

Finally, structural variations in NBCn2 might play a role in pathology. A latest study has shown that the molecular weight of NBCn1 is consistently higher in breast cancer tissues compared to that in normal tissues [34], suggesting a change in alternative splicing of SLC4A7 products, although it could also be due to a change in protein modification.

### Tissue specificity of alternative transcription of *Slc4a10*

Two studies have shown by northern blotting that NBCn2 transcripts are highly expressed in the brain, and less so in other tissues including the kidney, intestine, pituitary, and testis [10], [11]. The major portions of the probes used in these two studies correspond to the coding regions that are common in the transcripts encoding all NBCn2 variants. Thus, the signals of the northern blotting from these two studies should be representative of the total transcripts encoding all NBCn2 variants.

Our western blotting data show that NBCn2 proteins derived from the distal promoter P1 of *Slc4a10* are predominantly expressed in the brain and in much lower abundance in the eyes (Figure 10A). In contrast, those derived from the proximal promoter P2 of *Slc4a10* are predominantly expressed in the kidney, and to a lesser extent in the duodenum, jejunum, and ileum (Figure 10B). To our knowledge, our study is the first to show that NBCn2 proteins are expressed in tissues other than the CNS.

Our data suggest that the transcription of *Slc4a10* from alternative promoters is regulated in a tissue-specific manner. The distal promoter P1 primarily controls the expression of *Slc4a10* in the CNS (predominantly in the brain). In contrast, the proximal promoter P2 primarily controls the expression of *Slc4a10* in the kidney and the duodenum, jejunum as well as ileum.

Indeed, the transcription from alternative promoters is often regulated in a tissue specific manner. As far as other NCBTs are concerned, the epithelial cells of renal proximal tubules predominantly expresses NBCe1-A that is transcribed from the proximal promoter of *SLC4A4* [35]. On the other hand, many other epithelial tissues such as the pancreatic duct [36], [37], ameloblast [38], [39], and colonic epithelia [40] predominantly express NBCe1-B which is transcribed from the distal promoters of *SLC4A4*. The distal promoter expressing NBCe1-B contains a pH-responsive element that is responsible for the enhanced gene transcription activity upon acidic stimulus [39], a finding consistent with the up-regulation of NBCe1-B expression in ameloblast-like LS48 cells in response to acidic pH [38].

### Transcription activities of *Slc4a10* promoters in cultured HEK293 cells

We have shown that the expression of the alternative promoters of *Slc4a10* is highly tissue specific, i.e., the distal promoter P1 primarily in the brain vs. the proximal promoter P2 primarily in the kidney. A reasonable speculation is that promoter P2 should have a higher transcription activity than P1 should do in the cultured HEK293 cells, which is a human embryonic kidney cell line. However, our data indicate that the two promoters of *Slc4a10* have transcription activities of basically the same levels. One possible explanation is that the promoter sequences tested in the present study represent just the core regions of the two promoters that are sufficient to elicit basal transcription activity. It is possible that some yet unknown elements upstream of the core regions of the two promoters exist to enable the tissue specificity of the usage of alternative promoters. Also note that, HEK293 is an immortalized embryonic cell line so it may not be a perfect model of adult kidney.

### Tissue-type specificity of alternative splicing of *Slc4a10*

As shown in Figure 1A, *SLC4A10* gene contains two major alternative splicing units: insert A (exon 9) and insert B (exon 27). Omission of insert B results in the expression of NBCn2 variants with the long PDZ-Ct like those of NBCn2-C, -D, -G, and -H. In contrast, inclusion of insert B causes the expression of NBCn2 variants with the short non-PDZ-Ct. In the present study, we examined the expression specificity of the two types of NBCn2 with alternative Ct in two types of tissues: the neural tissues from the CNS vs. several selected epithelial tissues. Our data show that the expression of the two types of NBCn2 Ct arising from alternative splicing of insert B is highly tissue-type specific.

#### Choroid plexus in CNS

In CNS, the epithelial cells in the choroid plexus are responsible for the production of cerebrospinal fluid which contains high concentration of HCO_3_^−^. The epithelial cells in the choroid plexus express several members of NCBTs as well as other bicarbonate transporters mediating the transepithelial HCO_3_^−^ secretion (for review, see refs. [17]). NBCn2 plays a critical role, either direct or indirect, in the production of cerebrospinal fluid in the choroid plexus [5], [7].

Our RT-PCR analysis shows that insert B is absent in the *Slc4a10* transcripts expressed in the choroid plexus (Figure 8B). The full-length cDNA cloning data show that all NBCn2 variants identified from the choroid plexus contain the PDZ-Ct (Table 2), an observation that further confirms the absence of insert B in this tissue. We conclude that exon 27 of *Slc4a10* is not expressed in the choroid plexus from the CNS.

Note that, immunocytochemistry studies from several different groups have well established that NBCn2 is localized at the basolateral membrane of epithelial cells in the choroid plexus [5], [7], [12], [13], [16] (for review, see ref. [17]). Taking the previous immunocytochemistry results and our present data together, we conclude that the epithelial cells in the choroid plexus specifically express NBCn2 variants with the PDZ-Ct.

#### Other epithelial tissues

Similar to the choroid plexus in CNS, our RT-PCR analysis shows that insert B is also predominantly excluded in *Slc4a10* transcripts expressed in the kidney, male as well as female reproductive tract tissues (Figure 8B). Consistent with these observations, virtually all NBCn2 colonies identified from these tissues are NBCn2-C or NBCn2-G, with only one exception from the male reproductive tract tissues (Table 2). The epithelial cells in the kidney and reproductive tract tissues normally perform considerable HCO_3_^−^ transport (see review, see refs. [28], [41]). We hypothesize that, like the case of the choroid plexus, the epithelial cells in the kidney and the reproductive tract tissues specifically express NBCn2 variants with the PDZ-Ct.

#### Neural tissues in CNS

In contrast to the choroid plexus and other studied epithelial tissues, our RT-PCR analysis shows that insert B is predominantly included in the *Slc4a10* transcripts from the neural tissues including CX, HC, and OB (Figure 8B). Consistent with these observations, the vast majority of NBCn2 expressed in these neural tissues contain the non-PDZ-Ct (Table 2). Our data are consistent with a previous report by Giffard et al who have shown by RT-PCR that the vast majority of NBCn2 transcripts expressed in mouse and rat brain cortex contain insert B [15]. Note that, previous studies have demonstrated by immunocytochemistry that NBCn2 is expressed in neurons but not in astrocytes in the neural tissues [5], [12]. Taking the previous observations and our present data together, we conclude that the neurons in CNS predominantly, if not exclusively, express NBCn2 variants with the non-PDZ-Ct.

The very small fractions of NBCn2 variants (i.e., NBCn2-C/D/G/H) with PDZ-Ct in the neural tissues in Table 2 could arise from two sources. Firstly, it is possible that these NBCn2 variants are derived from the contamination of epithelial cells from the choroid plexus and/or the meninges. Secondly, we noted that, the aforementioned study by Giffard et al has shown the expression of NBCn2 transcripts lacking insert B from an astrocyte culture of rat [15]. On the other hand, two other groups have shown by immunostaining that NBCn2 protein is not detectable in either freshly dissociated mouse hippocampal astrocytes [12] or those from mouse brain sections [5]. Consider that the sensitivity of RT-PCR would be much higher than immunostaining with antibodies, it is possible that NBCn2 with PDZ-Ct (lacking insert B) is just expressed at very low abundance in a limited population of astrocytes. Thus, it is possible that the NBCn2 variants with PDZ-Ct in the neural tissues in Table 2 are derived from astrocytes.

Taking all together, we conclude that the alternative splicing of insert B in *Slc4a10* transcripts, therefore the expression of alternative NBCn2 Ct (PDZ-Ct vs. non-PDZ-Ct), is highly tissue-type specific. Moreover, it appears that the alternative splicing of insert B of *Slc4a10*, therefore the expression of alternative NBCn2 Ct, is cell-type specific, i.e., NBCn2 variants with the PDZ-Ct in epithelial cells vs. those with the non-PDZ-Ct in neurons.

Note that, due to the limited sampling in the colony screening in our study (i.e., the data in Table 2), it is not possible to completely rule out the expression of the NBCn2 with the short non-PDZ-Ct in the studied epithelial tissues. At least NBCn2-B (*aka* NCBE-B) with non-PDZ-Ct has been cloned from human kidney [42]. Nevertheless, in the epithelial tissues examined in the present study, NBCn2 with the non-PDZ-Ct, if any, must be expressed at extremely low abundance compared to the NBCn2 with PDZ-Ct.

### Implications for studying functional regulation of NBCn2

Sequence variations could provide the structural basis for differential regulation of the function of a transporter. For example, the alternative variants derived from the same gene may be subjected to the functional regulation via distinct signaling pathways in different tissues. Take NBCe1 as an example. In the renal proximal tubules, NBCe1-A is regulated by the insulin/angiotensin II signaling pathways (for review, see ref. [43]). In the pancreatic ducts, the activity of NBCe1-B is balanced by the regulation via the IRBIT/PP1 and the WNK/SPAK signaling pathways [44]. NBCe1-B is functionally up-regulated by IRBIT, an inositol-1,4,5-trisphosphate (IP3) receptor binding partner [45]. The stimulation effect of IRBIT on NBCe1-B depends on the presence of the first 16 amino-acid residues of NBCe1-B Nt, a portion unique compared to NBCe1-A [46].

Preliminary studies have shown that IRBIT can also enhance the activity of NBCn2-B when heterologously expressed in *Xenopus* oocytes [47], [48]. It will be worthy to study whether IRBIT has differential effects on NBCn2 variants with alternative Nt.

Lee et al [49] have shown that the activity of NBCn2-C (*aka* rb2NCBE) is inhibited by the activation of protein kinase A (PKA). This PKA-mediated inhibition on NBCn2-C relies on the interaction with ezrin binding protein EBP50 which contains a PDZ-domain. The study by Lee et al provides a functional relevance of the alternative NBCn2 Ct, a major difference in which is the presence/absence of the PDZ-binding motif. Our demonstration about the tissue-type specific (presumably even cell-type specific) expression of NBCn2 variants with alternative Ct provides insights into studying the molecular pathways underlying the functional regulation of the transporter. It is conceivable that the PKA/EBP50 dependent pathway involved in the regulation of NBCn2 is related to (and probably restricted to) the context of epithelial cells. It is also conceivable that the mechanism involved in the functional regulation of the non-PDZ-Ct NBCn2 expressed in neurons would be distinct from that involved in the regulation of the PDZ-Ct NBCn2 expressed in epithelial cells. In other words, the mechanisms underlying the functional regulation of alternative NBCn2 variants might be tissue-type (and even cell-type) specific.

### NBCn2 in kidney

The kidney is the organ responsible for the reabsorption of fluid as well as many ions in the renal filtrate. The reabsorption of fluid and ions is carried out by a series of transporters and channels expressed in the renal tubular epithelial cells.

Among the five NCBTs of SLC4 family, NBCe1 [35], [50], [51], NBCe2 [52], [53], NBCn1 [54], [55], and NDCBE [56] are all expressed in the kidney. These NCBTs are distributed in different segments of renal tubules and may play differential roles in maintaining the global acid-base homeostasis in the body. NBCe1-A is localized at the basolateral membrane of epithelial cells in the proximal renal tubules, playing an essential role in HCO_3_^−^ reabsorption in the kidney [35], [50], [51]. So far, 12 mutations in *SLC4A4* have been associated with proximal renal tubule acidosis in humans (for review, see refs. [28], [57]). NBCn1 is expressed at the basolateral membrane of epithelial cells in the thick ascending limb [54], [55]. NBCe2 [53] and NDCBE [56] are expressed at the collecting duct. Genetic disruption of *Slc4a5* causes metabolic acidosis with slightly decreased total [CO_2_] and increased HCO_3_^−^ secretion in urine [52].

A previous study has shown by northern blotting that *Slc4a10* transcripts are expressed in rat kidney [10]. Our western blotting data (Figure 10B) demonstrated that NBCn2 proteins with the novel Nt are most abundantly expressed in rat kidney. This observation suggests that NBCn2 play an important role in the renal acid-base transport of rat kidney. Immunocytochemistry study is necessary to further understand the physiological role of NBCn2 in the renal tubules. Nevertheless, our finding provides an important new perspective for studying the physiology of NBCn2 in the kidney.

Surprisingly, it appears that NBCn2 with the novel Nt is not expressed at adequate abundance in mouse kidney based upon the following two observations. Firstly, it has been extremely difficult to clone MQPG-NBCn2 from mouse kidney (see note c in Table 2). Secondly, we have also developed a polyclonal antibody against the first 15 aa (MQPGSCEHFQSLSQE) of mMQPG-NBCn2. Although this antibody specifically recognizes a GST fusion protein containing the novel Nt of mouse NBCn2 (data not shown), it is not able to recognize a band of expected molecular weight from kidney as well as other tissues of mice. Taken together, these observations suggest that, although rMCDL-NBCn2 (orthologs of mouse MQPG-NBCn2) is predominantly expressed in rat kidney, MQPG-NBCn2 may not be expressed in mouse kidney. More fine experiments are required to address the species difference regarding the expression of NBCn2 in the kidney.

### Summary and Conclusion

In the present study, we have made several major findings about NBCn2. Firstly, we have cloned for the first time six novel NBCn2 variants, the extreme Nt of which is distinct from that of the originally described NBCn2 variants. Secondly, we have demonstrated that *Slc4a10* contains two alternative promoters responsible for the expression of the two types of NBCn2 variants with distinct extreme Nt. Thirdly, we have shown that the expression of NBCn2 proteins with different extreme Nt exhibit distinct distribution patterns in rat tissues, suggesting that usage of the alternative promoters in *Slc4a10* expression is tissue-specific. Finally, we have demonstrated for the first time that the expression of NBCn2 with alternative Ct (PDZ-Ct vs. non-PDZ-Ct) is tissue-type specific, and likely cell-type specific. We conclude that the alternative transcription and alternative splicing of *Slc4a10* are regulated in a tissue-specific manner. These findings provide important insights into the study on the physiological roles of NBCn2, thus represent a major step forward towards understanding the molecular physiology of the transporter.
